# A positron emission tomography imaging study to confirm target engagement in the lungs of patients with idiopathic pulmonary fibrosis following a single dose of a novel inhaled αvβ6 integrin inhibitor

**DOI:** 10.1186/s12931-020-01339-7

**Published:** 2020-03-26

**Authors:** Toby M. Maher, Juliet K. Simpson, Joanna C. Porter, Frederick J. Wilson, Robert Chan, Rhena Eames, Yi Cui, Sarah Siederer, Simon Parry, Julia Kenny, Robert J. Slack, Jagdeep Sahota, Lyn Paul, Peter Saunders, Philip L. Molyneaux, Pauline T. Lukey, Gaia Rizzo, Graham E. Searle, Richard P. Marshall, Azeem Saleem, Arthur R. Kang’ombe, David Fairman, William A. Fahy, Mitra Vahdati-Bolouri

**Affiliations:** 1 Royal Brompton Hospital, London, UK; 2 National Heart and Lung Institute, Imperial College London, London, UK; 3 GlaxoSmithKline Research and Development, Stevenage, UK; 4 University College London Hospital, London, UK; 5 Invicro, A Konica Minolta Company, London, UK

**Keywords:** Alpha-v beta-6, αvβ6, Integrin, Idiopathic pulmonary fibrosis, IPF, positron emission tomography, PET, [^18^F]FB-A20FMDV2, target engagement

## Abstract

**Background:**

Idiopathic pulmonary fibrosis (IPF) is a chronic, progressive lung disease with poor prognosis and a significant unmet medical need. This study evaluated the safety, pharmacokinetics (PK) and target engagement in the lungs, of GSK3008348, a novel inhaled alpha-v beta-6 (αvβ6) integrin inhibitor, in participants with IPF.

**Methods:**

This was a phase 1b, randomised, double-blind (sponsor unblind) study, conducted in the UK (two clinical sites, one imaging unit) between June 2017 and July 2018 (NCT03069989). Participants with a definite or probable diagnosis of IPF received a single nebulised dose of 1000 mcg GSK3008348 or placebo (ratio 5:2) in two dosing periods. In period 1, safety and PK assessments were performed up to 24 h post-dose; in period 2, after a 7-day to 28-day washout, participants underwent a total of three positron emission tomography (PET) scans: baseline, Day 1 (~ 30 min post-dosing) and Day 2 (~ 24 h post-dosing), using a radiolabelled αvβ6-specific ligand, [^18^F]FB-A20FMDV2. The primary endpoint was whole lung volume of distribution (V_T_), not corrected for air volume, at ~ 30 min post-dose compared with pre-dose. The study success criterion, determined using Bayesian analysis, was a posterior probability (true % reduction in V_T_ > 0%) of ≥80%.

**Results:**

Eight participants with IPF were enrolled and seven completed the study. Adjusted posterior median reduction in uncorrected V_T_ at ~ 30 min after GSK3008348 inhalation was 20% (95% CrI: − 9 to 42%). The posterior probability that the true % reduction in V_T_ > 0% was 93%. GSK3008348 was well tolerated with no reports of serious adverse events or clinically significant abnormalities that were attributable to study treatment. PK was successfully characterised showing rapid absorption followed by a multiphasic elimination.

**Conclusions:**

This study demonstrated engagement of the αvβ6 integrin target in the lung following nebulised dosing with GSK3008348 to participants with IPF. To the best of our knowledge this is the first time a target-specific PET radioligand has been used to assess target engagement in the lung, not least for an inhaled drug.

**Trial registration:**

clinicaltrials.gov: NCT03069989; date of registration: 3 March 2017.

## Background

Idiopathic pulmonary fibrosis (IPF) is a specific form of chronic, progressive interstitial pneumonia of unknown cause [1, 2]. It occurs primarily in adults and the clinical course varies amongst diagnosed individuals from slow progression to acute decompensation and death, with a median survival time in untreated patients of just 2 to 4 years [1, 2]. Pharmacological treatment is currently limited to two oral anti-fibrotics, pirfenidone and nintedanib, both of which have been shown to reduce the rate of decline in lung function but are associated with significant side effects [2]. There remains a considerable unmet need for new therapies to treat this disease.

The alpha-v beta-6 (αvβ6) integrin has a major role in the activation of transforming growth factor-β (TGFβ), a cytokine that is central to the development of IPF [3]. The expression of αvβ6 is upregulated in IPF lung tissue [4], and increased levels of αvβ6 are associated with disease progression and poor prognosis [5], suggesting that an inhibitor of αvβ6 may provide therapeutic benefits to patients with IPF. GSK3008348 is a novel, small molecule αvβ6 integrin inhibitor that has been developed for inhaled delivery via nebulisation and is the first inhaled compound in this class [6]. In vitro, GSK3008348 demonstrates selective and high affinity binding to the αvβ6 integrin, inducing fast internalisation and degradation of the integrin. In an in vivo mouse model of lung fibrosis, inhaled GSK3008348 inhibited the activation of TGFβ [7]. GSK3008348 has previously been demonstrated to be well tolerated at single doses up to 3000 mcg in healthy human participants, with a pharmacokinetic (PK) profile that was dose proportional at potentially clinically relevant doses [8].

Positron emission tomography (PET) imaging using the αvβ6-selective [^18^F]FB-A20FMDV2 PET ligand provides a non-invasive method for measuring αvβ6 expression and a potential model for assessing target engagement of inhaled therapies in the lung [9, 10]. In a study of 15 participants (6 healthy, 9 with pulmonary fibrosis), lung uptake of [^18^F]FB-A20FMDV2 measured by lung volume of distribution (V_T_), a partition co-efficient that acts as a marker of expression of the integrin αvβ6, was successfully quantified. This demonstrated increased expression in IPF compared with healthy participants [10]. Additionally, [^18^F]FB-A20FMDV2 was well-tolerated and the PET measures were reproducible over a two-week period. An independent study has recently reported similar results with an alternative αvβ6-specific PET ligand ([^18^F]FP-R_0_1-MG-F2) [11].

The aim of this study was to evaluate the safety, tolerability and PK of GSK3008348 in participants with IPF and to obtain preliminary information on target engagement using the [^18^F]FB-A20FMDV2 PET ligand.

## Methods

### Study design

**Fig. 1:**
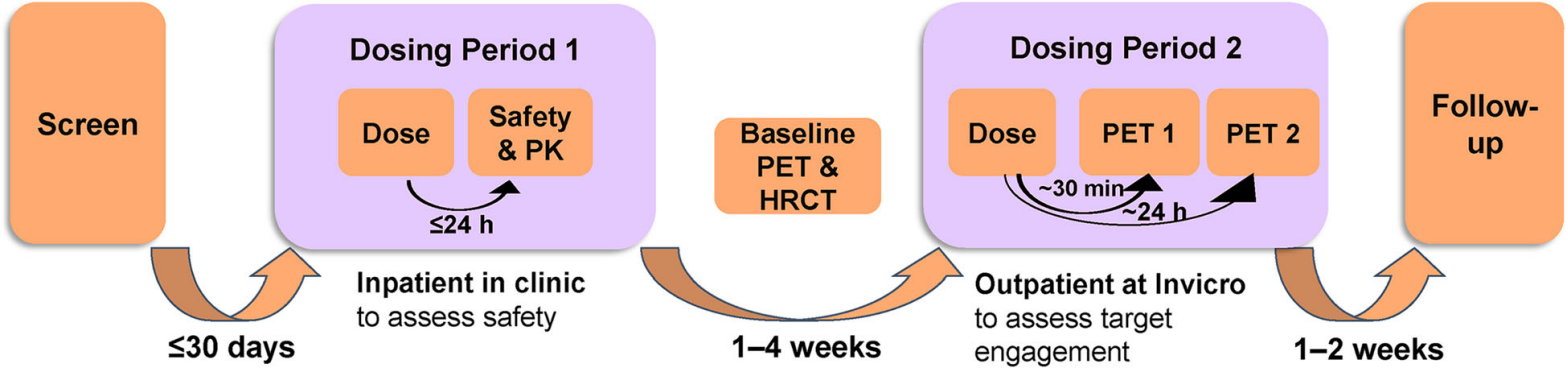
Study schematic for cohort 1. HRCT: high-resolution computed tomography; PET: positron emission tomography; PK: pharmacokinetics; PET1: PET scan on day 1 at ~30 min post-dose; PET2: PET scan on day 2 at ~24 h post-dose

This phase 1b study was conducted at three centres in the UK (two clinical sites [Royal Brompton Hospital and University College London Hospital, London, UK] and one imaging unit [Invicro, London, UK]) between June 2017 and July 2018. The study was designed to comprise up to two cohorts (cohort 2 was optional) (Study 204,715, NCT03069989). In cohort 1, under double-blind (sponsor unblind) conditions, participants were randomised 5:2 to receive a single nebulised dose of either 1000 mcg GSK3008348 or placebo in two dosing periods (Fig. 1).

Dosing period 1 was to assess safety and PK; participants remained in the clinical unit overnight to enable regular safety and PK assessments up to 24 h post-dose. Dosing period 2 was to enable assessment of target engagement. After a wash out period of 7 to 28 days following the first dose, participants attended the imaging unit on an outpatient basis for a pre-dose PET scan (pre-dose PET), to determine baseline levels of pulmonary uptake of the αvβ6-specific tracer, as well as a high-resolution computed tomography (HRCT) scan. Following the baseline scan, participants attended the clinical unit for safety assessments and once deemed fit to continue, attended the imaging unit for their second dose of study medication and post-dose PET scans: Day 1 [~ 30 min post-dosing (PET 1)] and Day 2 [~ 24 h post-dosing (PET 2)]. There was a maximum of 14 days between the baseline PET scan and the PET scan on Day 1.

Optional cohort 2 was to enable further exploration of the safety of GSK3008348 and/or target engagement if required. Following completion of cohort 1, no safety signals emerged that warranted further investigation and the pre-defined study success criterion for GSK3008348 target engagement was met. This, together with the operational complexities of the study, led to the decision to stop the study prior to progression to cohort 2.

To ensure consistency of nebulised dosing the same model of Philips Sidestream Plus nebuliser (Philips, Guildford, United Kingdom) was used at each site. The 1000 mcg dose was selected for two reasons;

1. It was lower than the maximum 3000 mcg dose used in healthy volunteers, which would enable significant safety margins in the event that systemic exposure was higher in IPF patients than healthy volunteers.
2. PK from the study in healthy participants [10], demonstrated peak systemic unbound concentrations at 1000 mcg that were significantly in excess of the in vitro dissociation constant (Kd). As lung tissue exposures are likely to be at least as high as the systemic exposure, it was expected that αvβ6 would demonstrate significant engagement at this dose.

The study was approved by the National Research Ethics Service Committee London and the Administration of Radioactive Substances Advisory Committee of the UK. Written informed consent was obtained from all participants.

### Participants

Eligible participants were male (aged ≥50 years) or female (aged ≥55 years) with a definite or probable diagnosis of IPF [12], forced vital capacity (FVC) > 50% predicted and diffusing capacity of the lungs for carbon monoxide (DLCO) > 40% predicted. Body weight was required to be ≥45 kg and body mass index within the range of 18.5 to 35.0 kg/m^2^. Participants who were currently taking pirfenidone or nintedanib (or who had received either within 30 days of the first dose of study treatment), had a current IPF exacerbation or respiratory tract infection, a post-bronchodilator forced expiratory volume in 1 s (FEV_1_)/ FVC ratio < 0.70, or known hepatic or biliary abnormalities were excluded. Full details of the eligibility criteria can be found on ClinicalTrials.gov (https://clinicaltrials.gov/ct2/show/NCT03069989).

### PET imaging assessments

Dynamic [^18^F]FB-A20FMDV2PET scans were acquired using a Siemens PET/CT system Biograph 6 TruePoint with TrueV or a Hi-Rez Biograph 6 (Siemens Healthcare, Erlangen, Germany). Full details of the methodology for image acquisition and analysis have been detailed elsewhere [10]. In brief, for each PET scan, [^18^F]FB-A20FMDV2 was injected into a cubital or forearm vein through an intravenous cannula and emission data were acquired for up to 90 min. An additional venous cannula was used to collect blood samples throughout the PET scan for quantification of the total and unmetabolized [^18^F]FB-A20FMDV2-related radioactivity in whole blood and plasma. These data were used alongside the images to derive an input function for the analysis of emission PET data based on a volume of interest (VOI) in the descending aorta. Using time activity curves from the whole lung VOI, PET outcome parameters of dynamic V_T_, a partition coefficient derived using a kinetic model, and static standardised uptake value (SUV), integrated from 10 to 30 mins post tracer administration, were obtained. Both V_T_ and SUV measures were expressed with and without correction for air volume – referred to as air-corrected V_T_ and SUV and uncorrected V_T_ and SUV from here on.

The primary pharmacodynamic endpoint was the change in the uptake of [^18^F]FB-A20FMDV2 in the whole lung, assessed as uncorrected V_T_ at ~ 30 min post-dose compared with pre-dose. V_T_ was chosen a priori as the appropriate measure to assess uptake of the αvβ6 PET ligand as it intrinsically accounts within the kinetic model for any distributional changes in the ligand, for example altered blood kinetics, that may be caused by the effects of a therapeutic αvβ6 inhibitor.

The total maximum effective radiation dose received per participant was estimated to be 20.9 mSv [13], comprised of three PET scans each with up to two low-dose computed tomography scans for attenuation correction, and a HRCT scan which was performed once only at baseline in dosing period 2 for anatomical localisation.

### Safety assessments

Full safety assessments were conducted at screening, pre-dose baseline and at regular intervals during each dosing period, including vital signs (blood pressure, heart rate, respiratory rate and temperature), 12-lead electrocardiograms (ECGs), routine safety laboratory tests and lung function. Oxygen saturation was measured continuously from pre-dose to 4 h post-dose in dosing period 1. Adverse events (AEs) were monitored throughout the study.

### Pharmacokinetic assessments

PK blood samples (2 mL) were collected at pre-dose, and at 15 and 30 min, 1, 2, 4, 8, 12, 18 and 24 h post-dose in dosing period 1, and on Day 1 (pre-dose, 15 and 30 min, 2 and 4 h post-dose) and Day 2 (on arrival and discharge at the imaging unit) in dosing period 2. Plasma samples were analysed for GSK3008348 using a validated analytical method based on protein precipitation extraction followed by liquid chromatography tandem mass spectrometry (LC-MS/MS) (Covance, Harrogate, UK). The lower limit of quantification for GSK3008348 was 50 pg/mL using a 50-μL aliquot of human plasma with an upper limit of quantification of 50,000 pg/mL.

### Statistical methods

A required sample size of five participants in the GSK3008348 group was based on a within-subject standard deviation in uncorrected V_T_ between two PET scans of 0.15 and a minimum detectable reduction of 6.2% [10]. The primary endpoint, change in uncorrected V_T_ at ~ 30 min post-dose compared with pre-dose, was analysed using a Bayesian repeated measures model assuming non-informative priors of the form N (mean = 0, SD = 1000) for all model parameters and a Toeplitz covariance matrix. The analysis was based on the PET data from the five participants who received GSK3008348. Placebo participants were included primarily as a comparator for safety assessments. PET data from the placebo participants were included on plots for reference but were not included in the analysis. The adjusted posterior median value of V_T_ ratio (PET 1/ Pre-dose PET) was calculated together with corresponding standard deviation and 95% highest posterior density (HPD) credible interval (CrI). The posterior probability that the true ratio PET1/Pre-dose is less than 1 (= true % reduction in V_T_ at ~ 30 min compared to Pre-dose > 0%) was calculated and the pre-defined study success criterion was defined as a posterior probability ≥80%. The same Bayesian model was used for the analysis of all other PET derived endpoints.

Safety data were summarised descriptively. GSK3008348 plasma concentration-time data were analysed using standard non-compartmental analysis (WinNonlin Version 7.0). Key parameters derived were: area under the concentration-time curve from time zero to t, where t is the time of the last quantifiable concentration (AUC_0-t_), maximum plasma concentration (C_max_) and time to C_max_ (T_max_).

## Results

### Participant characteristics

A total of eight participants were randomised, five to GSK3008348 and three to placebo; seven completed the study with a PET scan at ~ 30 min post dose (PET 1). Four participants (3 active, 1 placebo) underwent the ~ 24 h post dose scan (PET 2). One participant in the placebo group was withdrawn due to PET equipment failures as the post dose PET scans could not be rescheduled within the limits of the protocol. The majority of participants were male (7 of 8) with a mean age of 70 and 74 years in the placebo and GSK3008348 groups, respectively (Table 1). Median (range) percent predicted FVC was relatively well preserved in both groups (Placebo: 93% (78–94%); GSK300348: 91% (79–113%)) whilst median (range) DLCO was reduced (Placebo: 43% (43–50%); GSK300348: 48% (42–54%)).

**Table 1:** Demographic and baseline characteristics

	Placebo (***N*** = 3)	GSK3008348 1000 mcg (***N*** = 5)
**Age (years)**, mean (SD)	70.3 (3.1)	73.6 (2.3)
**Males**, n (%)	3 (100)	4 (80)
**BMI (kg/m**^**2**^**)**, mean (SD)	31.4 (1.1)	27.9 (1.9)
**Race**, n (%)		
White/Caucasian/European heritage	3 (100)	5 (100)
**Lung function**		
FVC percent predicted (%), median (range) FEV_1_ percent predicted (%), median (range) DLCO percent predicted (%), median (range)	92.6 (77.9–93.6) 90.0 (89.8–99.2) 43.3 (43.0–50.1)	91.0 (78.5–113.4) 99.0 (71.4–123.9) 48.0 (41.7–53.5)

*BMI* body mass index

### PET imaging results

**Fig. 2:**
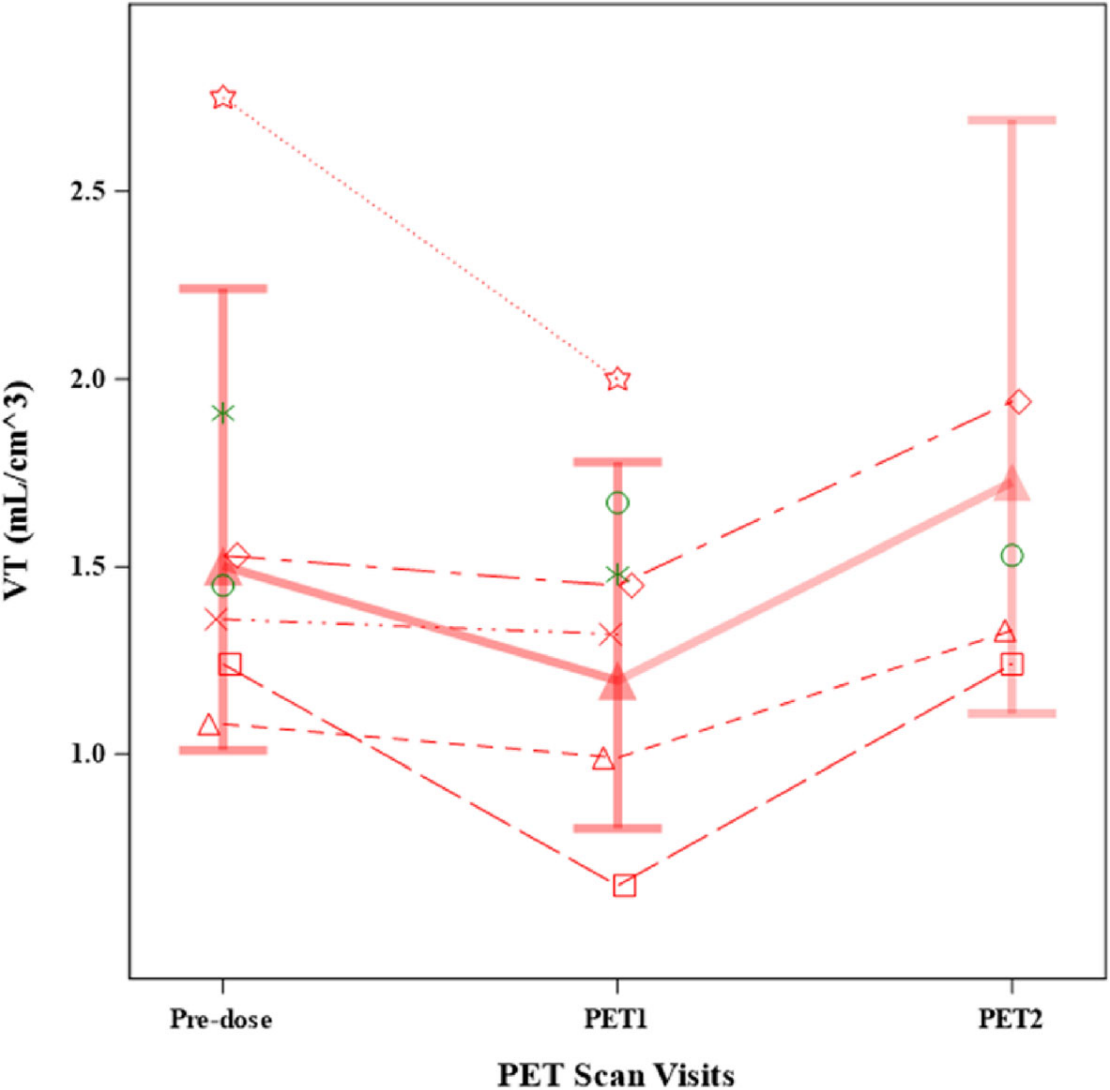
Summary of adjusted medians of uncorrected VT, (mL/cm3) of [18F]FBA20FMDV2 and individual patient profiles. PET1: PET scan on day 1 at ~30 min post-dose; PET2: PET scan on day 2 at ~24 h post-dose. The adjusted medians (with 95% Cr I) were calculated from a Bayesian repeated measures model using data from participants on the GSK3008348 1000 mcg arm only and is indicated by a thicker line and filled triangles. Data for individual participants dosed with GSK300834 are shown with dotted lines linking the data. Individual subject data is shown for participants on GSK3008348 1000 mcg (red symbols) and placebo (green symbols). The PET data from the placebo participants are included on plots for reference, but were not included in the analysis.

After single nebulised dosing with GSK3008348, adjusted median uncorrected V_T_ at ~ 30 min post-dose decreased from 1.50 mL/cm^3^ to 1.20 mL/cm^3^ and a reduction in uncorrected V_T_ was observed in all participants in the GSK3008348 group (Fig. 2; Additional File 1, Table S1). The adjusted posterior median reduction was 20% (95% HPD CrI: − 9 to 42%) and the posterior probability that the true % reduction at ~ 30 min post-dose compared with pre-dose > 0%, was 93%, meeting the pre-defined study success criterion (Table 2). The estimated standard deviation of log (PET1/Pre-dose) was 0.16 (Table 2). Three of the five participants receiving GSK3008348 had a PET scan at the ~ 24 h timepoint (PET 2) and for all three, uncorrected V_T_ increased from the ~ 30 min post-dose levels (Fig. 2) with a median adjusted V_T_ of 1.72 mL/cm^3^ at this time point (Additional File 1, Table S1). The estimated adjusted posterior median ratio of PET2/pre-dose was 1.154 (95% HPD CrI: 0.791, 1.647) and the posterior probability that the true % reduction at ~ 24 h post-dose compared with pre-dose > 0%, was 18% (Table 2).

**Table 2:** Adjusted posterior median ratios of uncorrected V_T_ (mL/cm^3^)

Parameter	Adjusted Median of Ratio	95% HPD CrI of Ratio	SD Logs of Ratio	Probability (True Ratio < 1.0)
**PET1/Pre-dose**	0.797	(0.578, 1.087)	0.1576	0.934
**PET2/Pre-dose**	1.154	(0.791, 1.647)	0.1779	0.175
**PET2/PET1**	1.446	(0.984, 2.083)	0.1868	0.030

*HPD Crl* Highest posterior density credible interval; *PET* positron emission tomography; *PET1* PET scan on day 1 at ~ 30 min post-dose (*n* = 5); *PET2* PET scan on day 2 at ~ 24 h post-dose (*n* = 3)

PET data from the placebo participants were included on plots for reference but were not included in the analysis

Data for air-corrected V_T_ showed a similar pattern to uncorrected V_T_ with a decrease in signal between PET1 and pre-dose (Additional File 2, Figure S1). However, the results for SUV (air-corrected and uncorrected) showed a different pattern to the V_T_ and did not show a decrease in signal between PET 1 and pre-dose (Additional File 3, Figure S2a and Figure S2b).

### Safety results

Six AEs were reported across four participants, three in the GSK3008348 group and one in the placebo group (Table 3). All AEs were mild in intensity and none were considered treatment related. There were no SAEs reported and no participant withdrawals from the study as a result of safety findings. Furthermore, no clinically significant abnormalities that were attributable to study treatment were reported for clinical laboratory evaluations, ECGs, vital signs or lung function assessments.

**Table 3:** Summary of all adverse events by treatment group

	Placebo (***N*** = 3)	GSK3008348 1000 mcg (***N*** = 5)
**Number of participants with any AE**, n (%)	1 (33)	3 (60)
Diarrhoea	1 (33)	0
Urinary tract infection	0	1 (20)
Headache	0	1 (20)
Contact dermatitis	0	1 (20)
Tooth extraction	0	1 (20)

### Pharmacokinetic results

**Fig. 3:**
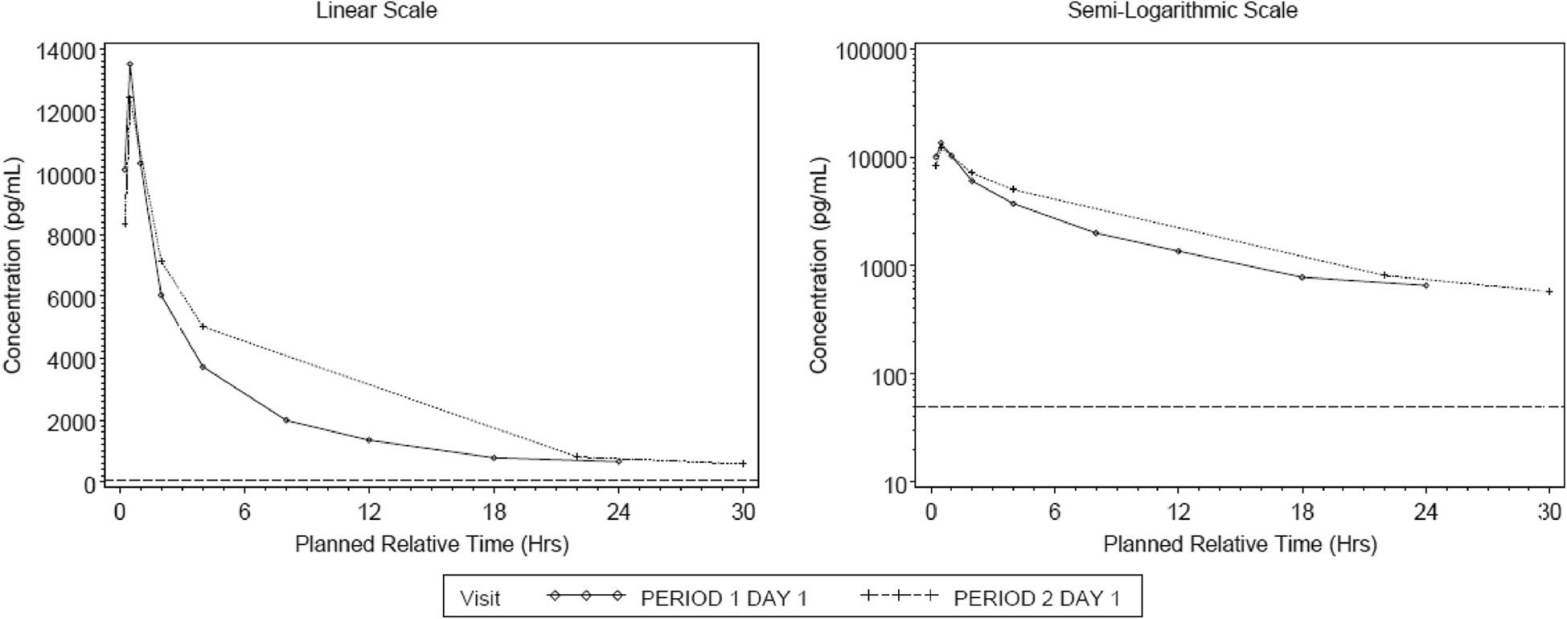
Median plasma GSK3008348 concentration-time plot

The plasma concentration-time profiles indicated rapid absorption of GSK3008348 (median T_max_ of 0.5 h) followed by a rapid decline in plasma concentrations up to 4 h post-dose and a slower decline thereafter (Fig. 3, Table 4). Plasma concentrations were measurable up to 24 h post-dose in period 1 and 30 h post-dose in period 2 with the exception of two participants whose last sampling time was 22 h. Systemic exposure to GSK3008348, as measured by geometric mean AUC_0-t_ and C_max_ did not appear markedly different between Dosing periods 1 and 2. Between subject variability (%CVb) in total systemic exposure (AUC_0-t_ and C_max_) to GSK3008348 was moderate (range 31 to 58%).

**Table 4:** Summary of Derived Plasma GSK3008348 Pharmacokinetic Parameters

PK Parameter (units)	N	Period	Geometric Mean	95% CI	%CVb
**AUC(0-t)** (h*pg/mL)	5	Period 1	64,638	(33,065, 126,362)	58
	5	Period 2	66,123	(39,036, 112,005)	44
**C**_**max**_ **(pg/mL)**	5	Period 1	14,050	(8876, 22,243)	38
	5	Period 2	10,418	(7191, 15,094)	31
**T**_**max**_^**1**^ **(h)**	5	Period 1	0.50	(0.4–0.5)	
	5	Period 2	0.47	(0.4–0.5)	

*AUC*_*0–t*_ area under the concentration-time curve from time zero to time of last quantifiable concentration; *C*_*max*_ maximum plasma concentration; *T*_*max*_ time to maximum plasma concentration

^1^T_max_ expressed as median (range)

## Discussion

This was the first study to evaluate the effects of the inhaled αvβ6 integrin inhibitor, GSK3008348, in an IPF population not currently treated with pirfenidone or nintedanib. The participants in this study were typical of an IPF population, 80% male and with a mean age > 70 years. The majority of participants had preserved FVC (median % predicted > 90%) but significantly impaired DLCO (median % predicted of 43.3% in the placebo group and 48% in the active treatment group) demonstrating the impact of fibrosis on gas transfer. A single nebulised 1000 mcg dose of GSK3008348 inhibited the uptake of the specific PET ligand [^18^F]FB-A20FMDV2 in the lung at ~ 30 min post-dose, providing evidence of target engagement with the αvβ6 integrin receptor, but not at ~ 24 h, indicating that the duration of target engagement was less than 24 h, consistent with a priori PK/PD modelling. Single doses of 1000 mcg of GSK3008348 were well tolerated, and PK was generally consistent with that observed for healthy volunteers [8].

All participants dosed with GSK3008348 showed a reduction in uncorrected V_T_, the primary endpoint, thus supporting the robustness of this finding. It is recognised that the treatment response differed between participants. It is difficult to explain the reasons for this in such a small study, but possible explanations include differences in drug exposure in the lungs and disease severity or activity. The variability (SD) of the data in the active group was 0.16, comparing favourably with the value of 0.15 assumed in the sample size calculations during study design, which were based on the test-retest variability measured in a previous enabling study, where PET scans were repeated ~ 12 days apart in patients with IPF [10].

The rationale for selecting uncorrected V_T_ as the primary endpoint was well considered when designing this study. IPF is characterized by interstitial pneumonia together with fibrosis and the distribution of air, fibrosis and blood in the lung varies according to the stage of fibrosis, all important factors to consider when interpreting lung [^18^F]FB-A20FMDV2 uptake [14]. V_T_ was chosen as the appropriate measure to assess the uptake of the αvβ6 PET ligand as, unlike SUV, it accounts for any distributional changes in the ligand that may be caused by a therapeutic αvβ6 inhibitor. This selection was corroborated as SUV indeed showed a different pattern to the V_T_ and did not decrease between PET 1 and pre-dose, strongly suggesting that distributional changes were relevant as expected.

The decision to use uncorrected rather than air-corrected V_T_ as the primary endpoint was based on the assumption that the fraction of air in the lung would be unchanged for an individual participant in this short-duration study. The relative changes in the post-dose air-corrected V_T_ were similar to those for uncorrected V_T_, justifying this assumption. As the reduction from pre-dose to ~ 30-min post-dose air-corrected V_T_ also met the pre-defined success criterion, this further supported the primary endpoint results.

The data presented here are insufficient alone to allow interpretation of how the 20% reduction in uncorrected V_T_ relates to the level of target engagement. However, putting these results into context, it is comparable to the 17% difference seen between IPF and healthy participants in air-corrected V_T_ [10], suggesting that it is a meaningful change. In making this comparison it is important to consider that some of the difference in PET signal between IPF and healthy participants will be due to reduced air fraction in IPF lungs, hence the difference in air-corrected V_T_ (which is smaller than the difference in uncorrected V_T_) is the appropriate comparator. In any case, the consistency in the relative change of uncorrected and air-corrected V_T_ values in the current study add further credence to the robustness of the result.

Data on uncorrected V_T_ at ~ 24 h post-dose, available for three out of five participants receiving GSK3008348, returned to approximate baseline levels - in line with a priori modelling that indicated the duration of action would be less than 24 h and hence not supporting a once daily dosing regimen for this drug. However, further study would be required to characterise the drug’s duration of action fully.

In this preliminary study in IPF patients, inhaled GSK3008348 was well tolerated with no AEs or clinically relevant findings attributable to treatment. The PK profile following single inhaled dosing demonstrated rapid absorption and multi-phasic elimination of GSK3008348. Furthermore, there was significant overlap in systemic exposure (C_max_ and AUC) to GSK3008348 in IPF patients compared with healthy participants following a 1000 mcg single dose [8].

## Conclusion

This first-time in patient IPF study successfully demonstrated that a nebulised 1000 mcg dose of GSK3008348 inhibited the uptake in the lung of a target-specific PET ligand for the αvβ6 integrin at ~ 30 min but not at ~ 24 h post-dose, providing evidence of target engagement and indicating a duration of target engagement of less than 24 h. Further study would be required to confirm the level of target engagement and to assess its impact on downstream pharmacology. GSK3008348 was well tolerated and PK was consistent with the PK profile shown in healthy subjects. To the best of our knowledge, this is the first time that a target-specific radioligand has been used to assess target engagement of a putative therapeutic compound in the lung, demonstrating proof of principle that PET imaging can enhance the understanding of pharmacology in early pulmonary drug development.

## Supplementary information

**Additional file 1: **Uncorrected Volume of Distribution VT; **Table S1.** Adjusted posterior median uncorrected VT (mL/cm^3^).

**Additional file 2: **Air-corrected V_T_; **Figure S1.** Summary of adjusted medians of air-corrected V_T_, (mL/cm^3^) of [^18^F]FB-A20FMDV2 and individual patient profiles

**Additional file 3: **Uncorrected and air-corrected Standardised Uptake Values (SUV); **Figure S2a.** Summary of adjusted medians of uncorrected SUV (g/mL) of [^18^F]FB-A20FMDV2 and individual patient profiles; **Figure S2b.** Summary of adjusted medians of air-corrected SUV (g/mL) of [^18^F]FB-A20FMDV2 and individual patient profiles

## Data Availability

Anonymized individual participant data and study documents can be requested for further research from: https://www.clinicalstudydatarequest.com

## Abbreviations

AE: Adverse event
αvβ6: Alpha-v beta-6
AUC_0–t_: Area under the concentration-time curve from time zero to time of last quantifiable concentration
BMI: Body mass index
CrI: Credible interval
C_max_: Maximum plasma concentration
CVb: Between subject coefficient of variation
DLCO: Diffusing capacity of the lungs for carbon monoxide
ECG: Electrocardiogram
FEV_1_: Forced expiratory volume in 1 s
FVC: Forced vital capacity
HRCT: High-resolution computed tomography
HPD: Highest posterior density
IPF: Idiopathic pulmonary fibrosis
Kd: Dissociation constant
LC-MS/MS: Liquid chromatography tandem mass spectrometry
PET: Positron emission tomography
PET1: Positron emission tomography scan on Day 1, ~ 30 min post-dosing
PET2: Positron emission tomography scan on Day 2, ~ 24 h post-dosing
PK: Pharmacokinetics
SUV: Standard uptake value
TGFβ: Transforming growth factor-β
T_max_: Time to maximum plasma concentration
VOI: Volume of interest
V_T_: Volume of distribution
