# Supplementary material for: A positron emission tomography imaging study to confirm target engagement in the lungs of patients with idiopathic pulmonary fibrosis following a single dose of a novel inhaled αvβ6 integrin inhibitor
Source: Respir Res. 2020 Mar 26;21:75. doi: 10.1186/s12931-020-01339-7 (PMC7099768; doi:10.1186/s12931-020-01339-7)
Supplement: Supplementary file 1 — Additional file 1: Uncorrected Volume of Distribution VT; Table S1. Adjusted posterior median uncorrected VT (mL/cm3). [file 12931_2020_1339_MOESM1_ESM.docx]

**Additional File 1**

**Uncorrected Volume of Distribution V_T_**

**Table S1: Adjusted posterior median uncorrected V_T_ (mL/cm^3^)**

| **N** | **Parameter** | **n** | **Adjusted Median** | **95% HPD CrI** | **SD Logs** |
| --- | --- | --- | --- | --- | --- |
| **5** | **Pre-dose** | 5 | 1.503 | (1.010, 2.240) | 0.2027 |
| **5** | **PET1** | 5 | 1.196 | (0.803, 1.779) | 0.2030 |
| **5** | **PET2** | 3 | 1.725 | (1.107, 2.691) | 0.2258 |

PET: positron emission tomography; PET1: PET scan on day 1 at ~30 min post-dose; PET2: PET scan on day 2 at ~24 h post-dose.

NB: PET data from the placebo participants were included on plots for reference but were not included in the analysis
