# Supplementary material for: A positron emission tomography imaging study to confirm target engagement in the lungs of patients with idiopathic pulmonary fibrosis following a single dose of a novel inhaled αvβ6 integrin inhibitor
Source: Respir Res. 2020 Mar 26;21:75. doi: 10.1186/s12931-020-01339-7 (PMC7099768; doi:10.1186/s12931-020-01339-7)
Supplement: Supplementary file 3 — Additional file 3: Uncorrected and air-corrected Standardised Uptake Values (SUV); Figure S2a. Summary of adjusted medians of uncorrected SUV (g/mL) of [18F]FB-A20FMDV2 and individual patient profiles; Figure S2b. Summary of adjusted medians of air-corrected SUV (g/mL) of [18F]FB-A20FMDV2 and individual patient profiles [file 12931_2020_1339_MOESM3_ESM.docx]

**Additional File 3**

**Uncorrected Standardised Uptake Values (SUV)**

The estimated adjusted posterior median uncorrected SUV showed an increase at ~30 min and ~24 h above baseline levels (Figure S2a). The estimated adjusted posterior median ratios were PET1/Pre-dose: 1.071 (HPD CrI: 0.910, 1.258), PET2/Pre-dose: 1.100 (HPD CrI: 0.926, 1.333) and PET2/PET1: 1.032 (HPD CrI: 0.849, 1.237). After conversion of the ratios the results were presented as a percentage (%) reduction. The estimated % reduction of uncorrected SUV at ~30 min was -7% (HPD CrI: -26% to 9%).

**Figure S2a: Summary of adjusted medians of uncorrected SUV (g/mL) of [^18^F]FB-A20FMDV2 and individual patient profiles**


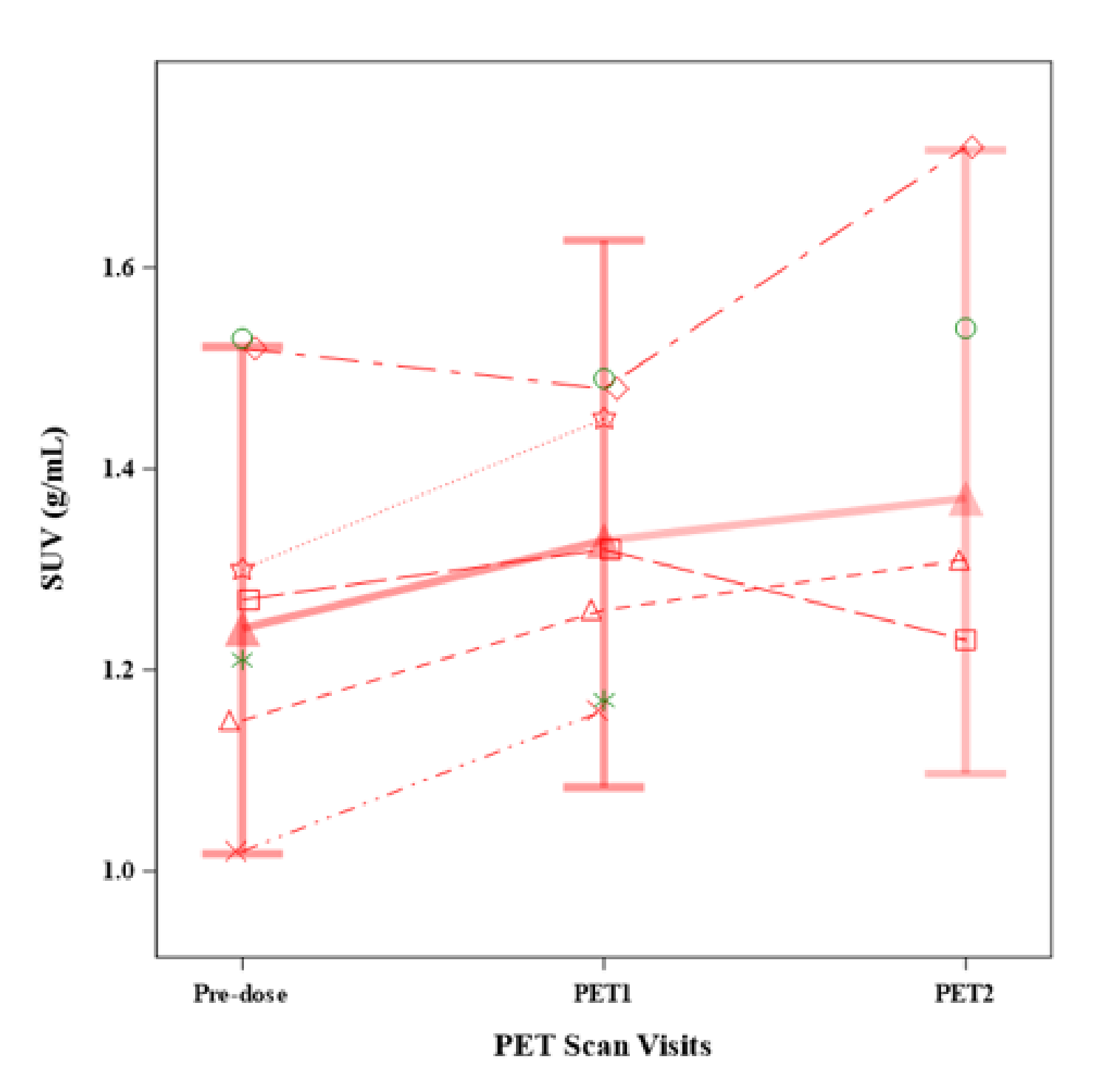


PET: positron emission tomography; PET1: PET scan on day 1 at ~30 min post-dose; PET2: PET scan on day 2 at ~24 h post-dose. The adjusted medians (with 95% Cr I) were calculated from a Bayesian repeated measures model using data from participants on the GSK3008348 1000 mcg arm only and is indicated by a thicker line and filled triangles. Data for individual participants dosed with GSK300834 are shown with dotted lines linking the data. Individual subject data is shown for participants on GSK3008348 1000 mcg (red symbols) and placebo (green symbols). The PET data from the placebo participants are included on plots for reference but were not included in the analysis.

**Air-corrected SUV**

The estimated posterior median air-corrected SUV showed an increase at ~30 min and then a slight decrease at ~24 h relative to 30 min post dose (Figure S2b). The estimated adjusted posterior median ratios were PET1/Pre-dose: 1.132 (HPD CrI: 0.971, 1.332), PET2/Pre-dose: 1.094 (HPD CrI: 0.904, 1.340) and PET2/PET1: 0.969 (HPD CrI: 0.801, 1.165). After conversion of the ratios the results were presented as a percentage (%) reduction. The estimated % reduction of air-corrected SUV at ~30 min was -13% (HPD CrI: -33% to 3%).

**Figure S2b: Summary of adjusted medians of air-corrected SUV (g/mL) of [^18^F]FB-A20FMDV2 and individual patient profiles**


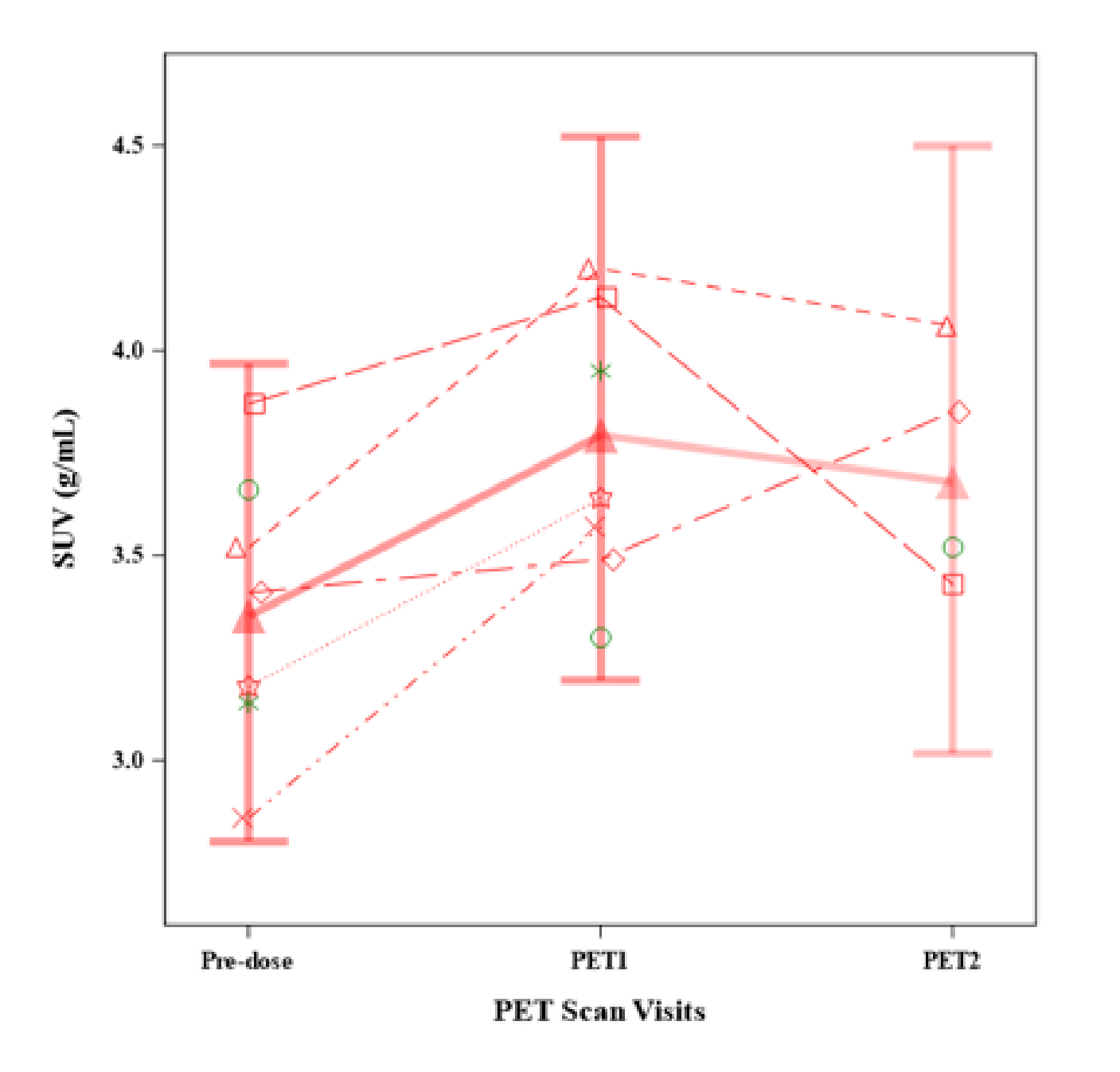


PET: positron emission tomography; PET1: PET scan on day 1 at ~30 min post-dose; PET2: PET scan on day 2 at ~24 h post-dose. The adjusted medians (with 95% Cr I) were calculated from a Bayesian repeated measures model using data from participants on the GSK3008348 1000 mcg arm only and is indicated by a thicker line and filled triangles. Data for individual participants dosed with GSK300834 are shown with dotted lines linking the data. Individual subject data is shown for participants on GSK3008348 1000 mcg (red symbols) and placebo (green symbols). The PET data from the placebo participants are included on plots for reference but were not included in the analysis.
